# Machine Learning for Predicting Venous Thromboembolism After Joint Arthroplasty: Systematic Review of Clinical Applicability and Model Performance

**DOI:** 10.2196/79886

**Published:** 2026-02-12

**Authors:** Junwei Ma, Huifeng Tang, Yunshan Zhang, Xuemei Yi, Tangsheng Zhong, Xinyun Li, Gang Wang

**Affiliations:** 1 School of Nursing, Jilin University, Changchun, China; 2 The Second Operation Room, First Hospital of Jilin University, No. 71 Xinmin Street, Chaoyang District, Changchun, China, 86 18186870822; 3 Nursing Department, First Hospital of Jilin University, Changchun, China

**Keywords:** joint arthroplasty, venous thromboembolism, machine learning, meta-analysis, systematic review

## Abstract

**Background:**

There is increasing research on machine learning in predicting venous thromboembolism after joint arthroplasty, but the quality and clinical applicability of these models remain uncertain.

**Objective:**

This systematic review aims to evaluate the predictive performance and methodological quality of machine learning models for venous thromboembolism risk after joint replacement surgery.

**Methods:**

Web of Science, Embase, Scopus, CNKI, Wanfang, Vipro, and PubMed were searched until December 15, 2024. The risk of bias and applicability were evaluated using the PROBAST (Prediction Model Risk of Bias Assessment Tool) checklist. A qualitative comprehensive analysis was conducted to extract and describe the data related to the model’s characteristics and performance.

**Results:**

This review encompassed 34 prediction models from 9 studies. The most frequently used machine learning models were extreme gradient boosting and logistic regression. The results showed that all studies had significant heterogeneity and high risk of bias. Although some models reported nearly flawless area under the curve (>0.9), they lacked external validation and may have overfitted. The models tested on large external datasets demonstrated more conservative performance.

**Conclusions:**

The predictive performance of machine learning models varied greatly. Although the reported area under the curve values indicated that some models have good discriminative ability, this performance varied greatly and was inconsistent among the included studies. These models have a high risk of bias, and it is necessary to take this into account when they are used in clinical practice. Future studies should adopt a prospective study design, ensure appropriate data handling, and use external validation to improve model robustness and applicability.

## Introduction

Joint replacement surgery is one of the main treatment methods for severe joint injuries, which can alleviate patients’ pain and improve joint function [1,2]. In recent years, due to the aging population and the advancement of medical technology, more and more patients have undergone joint replacement surgery. But surgical complications are a big risk that can change how well joint replacement surgery works. Venous thromboembolism (VTE), including deep vein thrombosis (DVT) and pulmonary embolism (PE), is a serious postoperative complication that significantly affects the prognosis of patients [3,4].

Studies have shown that for patients who did not receive preventive medication before undergoing major orthopedic surgeries, such as total hip arthroplasty (THA) or total knee arthroplasty (TKA), the incidence of postoperative VTE can be as high as 26.6% to 60.8% [5]. Asymptomatic VTE may not present any obvious clinical symptoms but can be detected through ultrasound examination or other diagnostic techniques. Some patients with VTE may experience discomfort and swelling in the affected limb, and their functional condition may be poor, which may even affect their postoperative recovery [6,7]. Furthermore, PE also has a significant impact on patients’ lives and prognosis. Studies have shown that up to 34% of postoperative deaths may be caused by PE [8,9].

Being able to accurately identify which patients are at high risk of VTE is of great significance for the prognosis of these patients. Accurate predictions can maximize the effectiveness of anticoagulant drugs and reduce side effects, such as bleeding [10,11]. Through the analysis of vast volumes of high-dimensional data, machine learning (ML) may uncover hidden patterns and intricate nonlinear relationships, aiding illness early warning [12,13]. In recent studies, ML models have been developed to predict the incidence of postoperative VTE. The most commonly used algorithms include extreme gradient boosting (XGB), random forest (RF), and support vector machine (SVM) [14]. In the prediction of VTE, ML technology exhibits many advantages. For instance, compared to traditional approaches, it can provide superior predictions by combining several types of information, such as imaging data, biomarkers, and clinical data [15,16]. Although these studies provide new methods for the field, there are some uncertainties in the consistency and reliability of the results in the existing literature owing to the single data source, small sample size, and the model’s limited capacity for generalization. In addition, there are justified concerns about bias, explainability, and applicability [17]. Therefore, this study aimed to assess the effectiveness and methodological quality of ML models in predicting VTE following joint replacement through a systematic review in order to provide evidence-based support for their clinical application, identify current research gaps, and offer guidance for future high-quality studies.

## Methods

### Registration of the Study

This study adheres to PRISMA (Preferred Reporting Items for Systematic Reviews and Meta-Analyses; Checklist 1) guidelines for conducting systematic reviews [18]. The study protocol was registered on PROSPERO (International Prospective Register of Systematic Reviews; registration number: CRD42024625842).

### Data Sources and Search Strategy

A comprehensive search was conducted in both Chinese and English databases, including Web of Science, Embase, Scopus, CNKI, Wanfang, Vipro, and PubMed, from the time of their formation until December 15, 2024. The following search terms were applied: “joint arthroplasty,” “venous thromboembolism,” “machine learning,” “risk prediction,” “prediction models,” and their synonyms and variant terms. A manual search of reference lists containing studies to find related publications was conducted. For detailed search strategies, see Multimedia Appendix 1, which includes keyword combinations and database settings.

### Inclusion or Exclusion Criteria

The following were the inclusion criteria: (1) studies on patients after joint replacement; (2) observational studies, case-control studies, cohort studies, and randomized controlled trials; (3) postoperative VTE risk was predicted using an ML model; (4) studies that provide performance evaluation metrics (eg, accuracy, sensitivity, specificity, area under the curve [AUC]) for the model and give clear statistical analysis results; and (5) studies published in English or Chinese.

The following were the exclusion criteria: (1) review articles, case reports, opinion pieces, commentaries, conference abstracts, and other nonoriginal research; (2) studies that do not mention particular kinds of ML models or algorithms; (3) animal experiments, simulation studies, and other nonclinical research; and (4) studies for which the entire text was not retrievable.

### Study Selection and Data Synthesis

The retrieved results were imported into EndNote 21, and duplicates were eliminated. Two researchers trained in systematic evaluation techniques independently conducted the study selection process based on preestablished eligibility criteria. First, the titles and abstracts of the literature were screened, and the studies failing to meet the eligibility criteria were initially excluded. Second, it was filtered through full-text reading, and if 2 researchers disagreed, a third researcher stepped in to reach an agreement.

The data extraction process was also performed independently by 2 researchers following CHARMS (Critical Appraisal and Data Extraction for Systematic Reviews of Prediction) guidelines [19], and a third researcher settled any discrepancies. There are 2 groups into which the data taken from the chosen studies are separated: (1) basic information: author, year of publication, country, study design, participants, data source, clinical outcome, and sample size and (2) model information: missing data handling, candidate predictors, screening variable methods, prediction timepoint, prediction horizon, model development, internal verification, external verification, model performance, number of variates, calibration, and method of visualization.

### Risk of Bias Assessment

The included studies’ applicability and bias risk were evaluated using the PROBAST (Prediction Model Risk of Bias Assessment Tool) tool. The assessment has 20 questions, including 4 basic areas: participants, predictors, outcomes, and analysis. There are answers to the questions in each domain: “yes/probably” (low risk of bias), “no/probably not” (high risk of bias), and “no information” (unclear). A domain was deemed to have a high risk of bias if its answer contained “no/probably not.” If every question had a “yes/probably yes” response, the domain was deemed to have a low risk of bias. When there is “no information,” it is deemed to be highly biased if it cannot be explained by the original or additional material. If each domain was considered low risk, then the total bias risk was considered low. Furthermore, the total risk of bias was deemed high if any domains were deemed to be at high risk.

The bias risk in each model is assessed independently by 2 researchers, and if there are differences, they will be discussed to reach an agreement. Due to the majority of research using various models or having several outcomes, the bias risk is evaluated independently for each model and outcome. Finally, since there are no differences in the risk of bias between models in a single study, each study only shows the result once.

## Results

### Results of Literature Screening

**Figure 1.**
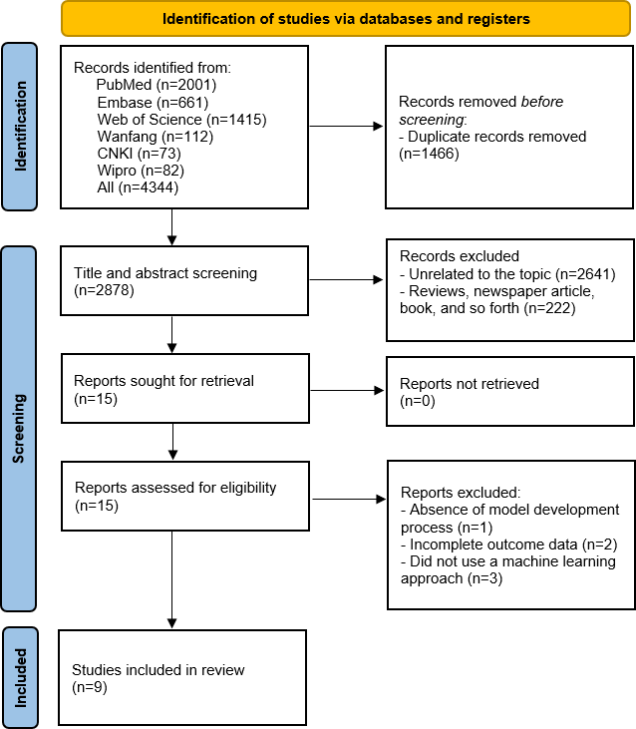
Literature screening process. CNKI: China National Knowledge Infrastructure.

The study selection process is detailed in the PRISMA flow diagram (Figure 1). A total of 4344 records were initially identified from 6 electronic databases (PubMed, n=2001; Embase, n=661; Web of Science, n=1415; Wanfang, n=112; CNKI, n=73; and Wipro, n=82). After removing 1466 duplicates, 2878 unique records remained for screening. During the title and abstract screening phase, 2863 records were excluded because they were either unrelated to the topic (n=2,641) or were nonoriginal research, such as reviews and newspaper articles. This left 15 reports to be sought for full-text retrieval. The subsequent critical appraisal excluded 6 studies based on predefined criteria: 3 did not use an ML approach, 2 had incomplete outcome data, and 1 did not involve a model development process. Ultimately, 9 studies met the full inclusion criteria and were included in this systematic review.

### General Study Characteristics

Table 1 shows the basic characteristics of the 9 studies. These studies were published from 2018 to 2024 and included 1 prospective study [3] and 8 retrospective studies [20-27]. Among them, 4 studies [3,20,21,25] were conducted in China; 3 studies [22-24] were from the United States; 1 was conducted in the Netherlands; and 1 transnational study used data from the Netherlands, the United Kingdom, and Denmark. In terms of study population, 2 studies [3,20] focused on patients with THA, 1 study [21] targeted patients with TKA, and the remaining studies [22-27] focused on both THA and TKA. In terms of clinical outcomes, 2 studies [20,21] concentrated solely on DVT, while the remaining 7 studies [3,22-27] evaluated both DVT and PE. In addition, the studies’ sample sizes ranged from 60 to 3,92,661. Cases of VTE ranged from 24 to 4042, with an incidence of 0.9% to 40%.

**Table 1.** Basic characteristics of the included studies.

Author (year)	Country	Research type	Data source	Participants	Clinical outcome	Cases/sample size
Ding et al (2023) [3]	China	Prospective study	Shanghai Changzheng Hospital	THA^a^	VTE^b^	76/1481
Chen and Jiang (2022) [21]	China	Retrospective study	Clinical database of a hospital in Chongqing	TKA^c^	DVT^d^	24/60
Harris et al (2019) [22]	United States	Retrospective study	VASQIP data, VA Corporate Data Warehouse	TKA, THA	VTE	566/70,569
Rasouli Dezfouli et al (2022) [23]	United States	Retrospective study	The Cerner Health Facts data warehouse	TKA, THA	VTE	4042/392,661
Shohat et al (2023) [24]	United States	Retrospective study	Rothman Orthopedic Institute at Thomas Jefferson University	TKA, THA	DVT, PE^e^	308/35,963
Wang et al (2023) [25]	China	Retrospective study	A health system	TKA, THA	VTE	1161/6897
Xu et al (2024) [20]	China	Retrospective study	A hospital in Wenzhou, China	THA	DVT	92/333
Sweerts et al (2022) [26]	Netherlands	Retrospective study	Multiple databases from the Dutch	TKA, THA	VTE	26/3776
Nemeth et al (2024) [27]	Netherlands, Denmark, England	Retrospective study	Multiple databases from the United Kingdom, the Netherlands, and Denmark	TKA, THA	VTE	897/64,032

a THA: total hip arthroplasty.

b VTE: venous thromboembolism.

c TKA: total knee arthroplasty.

d DVT: deep vein thrombosis.

e PE: pulmonary embolism.

### Characteristics and Performance of ML Models

**Figure 2.**
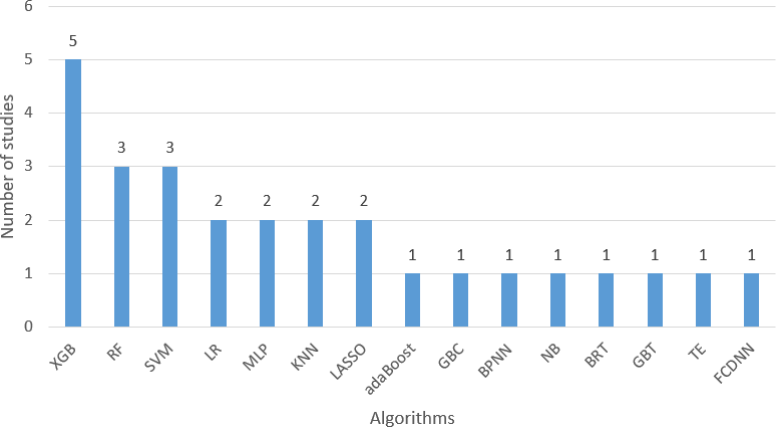
Frequency of machine learning models. adaBoost: adaptive boosting; BPNN: back propagation neural network; EM: ensemble method; FCDNN: fully connected deep neural network; GBT: gradient boosting tree; GBC: gradient boosting classifier; KNN: k-nearest neighbors; LASSO: least absolute shrinkage and selection operator regression; LR: logistic regression; MLP: multilayer perceptron; NB: naive Bayes; RF: random forest; SVM: support vector machine; TE: tree-based ensemble methods; XGB: extreme gradient boosting.

Table 2 provides a detailed summary of the methodological design and data characteristics of the included studies. Table 3 presents a summary of the performance of the model development, validation, and inclusion of the studies. Among the 9 studies included, 5 studies [3,20,23-25] evaluated multiple ML algorithms, and 4 studies [21,22,26,27] used a single algorithm. Across these studies, 15 kinds of ML algorithms were used to develop 34 predictive models, and each study developed 1-5 models. Logistic regression (LR) and XGB were the most common algorithms, appearing in 5 studies (5/9, 56%) each. This is followed by RF (3/9, 33%) [23-25] and SVM (3/9, 33%) [20,24,25]. In particular, the XGB model showed strong performance in predicting DVT and PE, with an AUC range of 0.71‐0.982. Figure 2 details the frequency of the model used.

**Table 2.** Summary of methodological design and data characteristics of the included studies.

Author (year)	Missing data handling	Candidate predictors	Screening variable methods	Prediction timepoint	Prediction horizon	Number of variates
Singh et al (2019) [1]	Data were excluded, or mean imputation, or mode imputation	67	LASSO^a^ regression	Preoperative	During postoperative hospitalization	27
Yau et al (2022) [2]	NR^b^	NR	Single-factor analysis	Postoperative	Within 3-5 days after surgery	8
Ding et al (2023) [3]	Treats missing data as a separate category	NR	NR	Preoperative	Within 30 days after surgery	22
Simon et al (2023) [4]	NR	NR	Genetic algorithm	Preoperative	Within 30 days after surgery	25
Lee et al (2013) [5]	NR	56	Univariate analysis	Postoperative	Within 90 days after surgery	27
Malcolm et al (2020) [6]	NR	66	NR	Preoperative	Within 5 weeks after surgery	66
Lewis et al (2019) [7]	missForest	26	Recursive feature elimination	Postoperative	During postoperative hospitalization	10
Meng et al (2021) [8]	Multiple imputation	5	Literature, clinical reasoning, eyeballing	Preoperative	Within 1 year after surgery	5
Runner et al (2021) [9]	No missing data	39	LR^c^	Preoperative	Within 90 days after surgery	12

a LASSO: least absolute shrinkage and selection operator regression.

b NR: not reported.

c LR: logistic regression.

**Table 3.** Summary of model development, validation, and performance of included studies.

Author (year)	Model development	Internal verification	External verification	AUC^a^ (95% CI)	Calibration	Method of visualization
Singh et al (2019) [1]	MLP^b^, XGB^c^, AdaBoost^d^, GBC^e^, KNN^f^, LR^g^	10-fold cross-validation, random split validation	None	0.955 (0.917‐0.993); 0.982 (0.954‐1.000); 0.980 (0.944‐1.000); 0.978 (0.953‐1.000); 0.837 (0.743‐0.931); 0.944 (0.878‐1.000)	NR^h^	ROC^i^ curve, DCA^j^ curve, Shapley additive explanations, nomogram
Yau et al (2022) [2]	XGB	Random split validation	None	0.832 (0.748‐0.916)	NR	ROC curve
Ding et al (2023) [3]	LASSO^k^	Internal cross-validation	Yes	0.613 (0.608‐0.617)	Calibration plot	Calibration curve
Simon et al (2023) [4]	RF^l^, GBT^m^, TE^n^, FCDNN^o^	Random split validation	None	0.698; 0.638; 0.635; 0.780	NR	ROC curve
Lee et al (2013) [5]	XGB, RF, LASSO, SVM^p^	Random split validation, repeated cross-validation	None	DVT^q^: 0.759 (0.667‐0.881); 0.71; 0.68; 0.64; PE: 0.774 (0.683‐0.835); 0.8; 0.8; 0.67	Calibration plot	ROC curve, PR^r^ curve, calibration curve
Malcolm et al (2020) [6]	XGB, RF, SVM, BPNN^s^, LR, EM^t^	5-fold cross-validation, GridSearchCV	None	0.914 (0.894‐0.935); 0.907 (0.883‐0.931); 0.903 (0.874‐0.930); 0.910 (0.8949‐0.926); 0.885 (0.868‐0.902); 0.921 (0.896‐0.936)	NR	ROC curve
Lewis et al (2019) [7]	XGB, KNN, SVM, NB^u^, MLP^v^, LR	Random split validation, 10-fold cross-validation	None	0.800 (0.674‐0.927); 0.755 (0.634‐0.874); 0.753 (0.627‐0.878); 0.681 (0.571‐0.788); 0.739 (0.611‐0.846); 0.579 (0.450‐0.696)	Brier score	Shapley additive explanations, feature importance
Meng et al (2021) [8]	Multiple imputation	LR	None	0.663 (0.627‐0.699)	Hosmer-Lemeshow, calibration plot, Brier score	ROC curve, calibration curve
Runner et al (2021) [9]	No missing data	LR	Yes	0.65 (0.63‐0.67) external validation: 0.64 (0.61‐0.67)	Calibration plot	Calibration curve

a AUC: area under the curve.

b MLP: multilayer perceptron.

c XGB: extreme gradient boosting.

d AdaBoost: adaptive boosting.

e GBC: gradient boosting classifier.

f KNN: k-nearest neighbors.

g LR: logistic regression.

h NR: not reported.

i ROC: receiver operating characteristic.

j DCA: decision curve analysis.

k LASSO: least absolute shrinkage and selection operator regression.

l RF: random forest.

m GBT: gradient boosting tree.

n TE: tree-based ensemble methods.

o FCDNN: fully connected deep neural network.

p SVM: support vector machines.

q DVT: deep vein thrombosis.

r PR: precision-recall.

s BPNN: back propagation neural network.

t EM: ensemble method.

u NB: naive Bayes.

v MLP: multilayer perceptron.

The included studies used a variety of metrics to evaluate ML models, of which AUC was the most common (8/9, 89%). The predicted AUC range for DVT and PE was 0.579‐0.982. One study did not report AUC but reported C-statistic [22]. XGB, popularized by Chen and Guestrin [28] in 2016, is a widely adopted ML algorithm on the basis of gradient boosting [29,30]. The included XGB studies [3,20,21,24,25] reported AUC ranging from 0.71 to 0.982, demonstrating strong model performance. LR estimates the association of one or more independent variables with binary dependent variables [31,32]. The included LR studies [3,20,25-27] reported AUC ranging from 0.579 to 0.944. RF is a widely used ML algorithm that helps mitigate overfitting and improves the model’s stability and accuracy by aggregating the outcomes from various decision trees [33]. It functions as a modeling algorithm as well as a variable selection technique. Three studies [23-25] used RF, with reported AUC ranging from 0.698 to 0.907. In addition, calibration plots were provided in 3 studies [22,24,27]; Brier scores were provided in 1 study [20]; and 1 study [26] also provided Hosmer-Lemeshow, calibration plot, and Brier score. The other 4 studies did not include data on calibration performance. Additional reported metrics encompass accuracy, sensitivity, and specificity.

These 9 studies differ in terms of the optimal timing for risk assessment. Six studies have created preoperative prediction models [3,22,23,25-27] to identify high-risk individuals before surgery. The last 3 studies focused on postoperative prediction, assessing immediate risks using postoperative data [20,21,24]. Additionally, there is diversity in the risk assessment’s predictive horizon. Its duration ranges from a brief period following the procedure, such as “within 3‐5 days after surgery” or “during the postoperative hospital stay” [3,20], to a lengthy risk assessment that might continue for up to a year following the procedure [26]. Common time horizons include 30 days [22,23] and 90 days [24,27].

**Figure 3.**
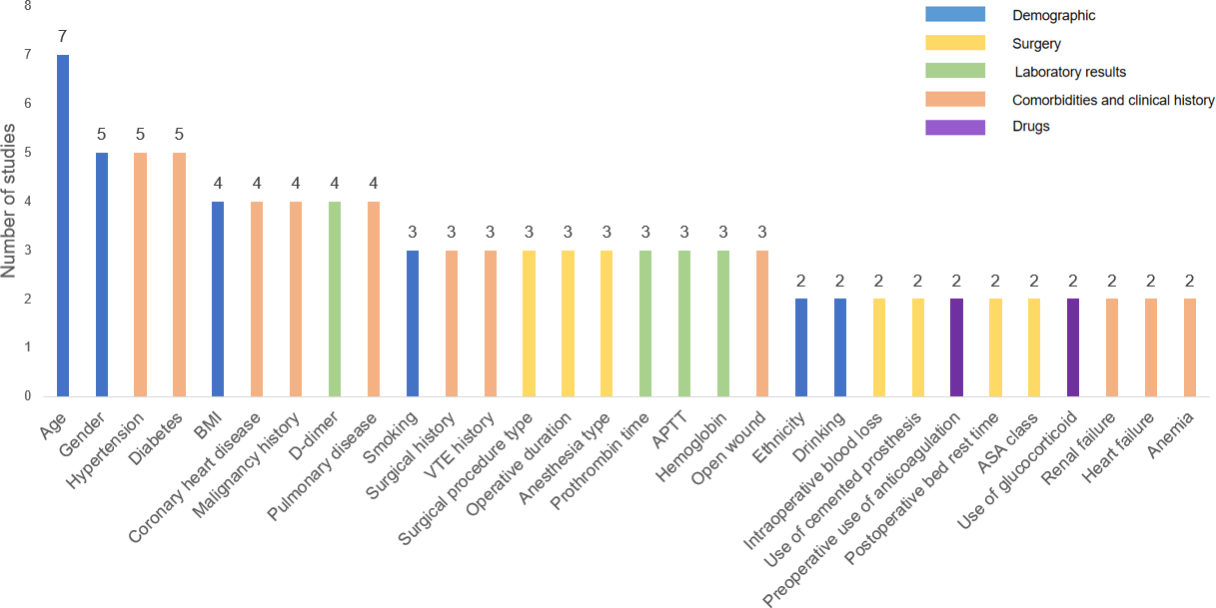
Frequency of the predictors. APTT: activated partial thrombin time; ASA: American Society of Anesthesiologists; VTE: venous thromboembolism.

From a low of 5 [26] to a high of 66 [25], the number of predictor variables that ultimately made it into the model varied widely. The descriptive analysis of the predictive factors used in each study (as shown in Figure 3) shows that a constant core set of variables is regularly utilized, despite the wide variations in the number of predictive factors used in each investigation. These variables can be broadly classified into four categories: (1) demographic and biological characteristics of the patients (such as age, gender, BMI); (2) major comorbidities (such as hypertension, diabetes, coronary heart disease, and malignant tumors); (3) specific VTE risk histories (such as previous VTE); and (4) key laboratory indicators (such as D-dimer).

### Model Validation

All of the models in the included studies underwent internal validation; however, only 2 studies [22,27] used external validation and reported the AUC value of external validation. The main methods used for internal validation were k-fold cross-validation and random-split validation. External validation plays a key role in confirming whether a model can maintain good performance in different populations and clinical settings. The absence of external validation will limit the generalization and clinical application of these models.

### Assessing Risk of Bias

According to the PROBAST evaluation, all models had a high risk of bias (Table 4). The assessment results showed that the main sources of bias were concentrated in the “participants” and “analysis” domains. In the participants domain, 8 studies had a high risk of bias, mainly because these studies relied on retrospective data. In the analysis domain, all models had a high risk of bias. The PROBAST assessment pointed out several specific methodological issues: 4 studies [21,23-25] did not perform model calibration, 4 studies [3,21,23,25] did not report the handling of missing data, and 2 studies [21,23] were insufficient in measures to prevent model overfitting. In contrast, the outcome domain was rated as having a low bias risk level in all studies. In terms of applicability assessment, only 1 study [25] had poor applicability because the model was overly complex and required 66 predictor variables.

**Table 4.** Prediction Model Risk of Bias Assessment Tool (PROBAST) results of the included studies.

Author (year)	Risk of bias				Applicability			Overall	
	Participants	Predictors	Outcome	Analysis	Participants	Predictors	Outcome	Risk of bias	Applicability
Ding et al (2023) [3]	+^a^	+	+	–^b^	+	+	+	–	+
Chen et al (2023) [21]	–	+	+	–	+	+	+	–	+
Harris et al (2019) [22]	–	+	+	–	+	+	+	–	+
Rasouli et al (2022) [23]	–	+	+	–	+	+	+	–	+
Shohat et al (2022) [24]	–	+	+	–	+	+	+	–	+
Wang et al (2023) [25]	–	–	+	–	+	–	+	–	–
Xu et al (2024) [20]	–	+	+	–	+	+	+	–	+
Sweerts et al (2022) [26]	–	+	+	–	+	+	+	–	+
Nemeth et al (2024) [27]	–	+	+	+	+	+	+	–	+

a Low risk of bias.

b High risk of bias.

## Discussion

### Principal Findings

VTE is a common and serious postoperative complication that is preventable [10]. Risk assessment and management-based strategies can help prevent or reduce the occurrence of VTE [34]. Therefore, selecting an appropriate predictive model for VTE is crucial. This systematic review examined the methodological quality and clinical applicability of ML methods in predicting VTE after joint replacement surgery. Among the reviewed studies, 15 ML algorithms were utilized to construct 34 predictive models. XGB and LR were the most used model types, followed by RF and SVM. The AUC values of the models ranged from 0.579 to 0.982, indicating that the discrimination ability of the models varied greatly, and some of the models showed excellent performance. Two research [1,6] reports demonstrated extremely high discrimination ability, with their AUC values exceeding 0.9. Such high performance often raises concerns about overfitting, where the model learns specific statistical noise specific to the training data rather than the generalizable underlying biological signals, leading to an optimistically biased performance estimate. Moreover, these models have not yet undergone external validation. This deficiency is a major limitation. In the clinical environment of joint replacement, there may be significant differences among patients, and surgical techniques as well as VTE prevention protocols may also vary among different medical institutions. Models trained based on data from a single hospital are likely to learn the specific treatment patterns of that hospital rather than a universally applicable VTE prediction signature. This may lead to unreliable prediction results when the model is applied to different patient groups. The risk of overfitting and the lack of reliable external validation seriously undermine the clinical value of these highly performing models. These models can only be considered for practical application after undergoing extensive external validation.

Another finding is that the XGB model demonstrates superior predictive performance compared to other models. For instance, 2 studies [3,20] compared the XGB and SVM models with the LR models, and the results showed that the XGB and SVM models outperformed the LR model. This supremacy is probably due to the smart way the algorithm was made. First, XGB has strong built-in features (such as regularization) that keep the model from overfitting when working with small datasets, making it stable even when the data are noisy. Second, XGB is very good at identifying the most important risk factors on its own. It can also capture complicated nonlinear interaction relationships, which typical linear models cannot do. Furthermore, the fact that XGB functions so effectively most likely indicates that the research team spent a great deal of time and energy optimizing the hyperparameters and determining the ideal configurations for their particular dataset. However, research has indicated that LR models may be more successful and easier to apply in some situations, whereas XGB and SVM models may not necessarily perform better. LR’s fundamental structure makes it simple to comprehend and analyze. Additionally, it produces dependable findings and has strong convergence, particularly when used for small datasets [35]. The Caprini score is a clinically prevalent instrument utilized to evaluate VTE risk by analyzing many clinical risk factors, including age, surgical history, malignancy, and obesity [36]. One study [25] compared the Caprini Score to ML models and showed that the ML models were better at predicting VTE risk. Although the predictive performance of most models ranged from moderate to good, the risk of bias in all studies restricted the applicability of these models in clinical practice.

### Related Work

The PROBAST assessment results indicate that all the models have a high risk of bias, suggesting that their predictive performance in practical applications may be lower than the reported levels. This widespread bias is mainly due to the flaws in the research design and the statistical analysis methods adopted by the models. In terms of the research design, most studies rely on retrospective data, which increases the risk of selecting an unrepresentative patient population, thereby weakening the generalization ability of the models beyond the original dataset. Additionally, there are numerous flaws in the analysis aspects of the model construction and testing processes. The main issues are that some studies did not check the calibration process. This makes it impossible for us to determine whether the “30% risk of venous thrombosis” predicted by the model truly means a 30% probability in reality, thus preventing its use in clinical decision-making. Some studies also failed to provide enough protections against the “overfitting” problem, in which the model just remembered the noise in the training data instead of identifying the true risk patterns, while others did not provide solutions for handling missing patient data. These analytical flaws make these models likely to perform poorly when applied to new patients. Furthermore, the actual usability is also an issue. One model used an excessive 66 predictive variables, making it overly complex and unsuitable for clinical use. To raise the accuracy and clinical application of predictive models, future studies should apply more rigorous methodological methods. Moreover, the significant bias risk indicates inconsistency in the development and validation of current models, which could influence their widespread application. Future studies should give priority to enhancements in a number of important domains, including the research design (ie, cohort or case-control study design), the number of events, the method for predictor selection, the processing of complicated data, and the processes of model calibration and fitting.

One serious and widespread flaw in these studies is the absence of model calibration reports. Although most of the included studies reported high discrimination metrics such as AUC, the discrimination does not guarantee that the predicted probabilities are accurate. Calibration addresses a more fundamental clinical question: Does the predicted “30% risk of venous thrombosis” really mean that there will be a 30% incidence rate among a group of similar patients? Inaccurate calibration might have very harmful consequences. For example, a model that consistently overestimates risks may incorrectly identify patients with low risks as having high risks [37]. This may lead to the improper use of potent anticoagulant drugs, thereby exposing patients to a serious risk of bleeding, a risk that could have been avoided. However, if the model underestimates the risks, it may prevent doctors from taking necessary preventive measures to ensure the safety of patients with high risk. This could lead to potentially fatal thrombosis incidents [38]. Consequently, owing to the absence of calibration data, most of these models are unreliable for informing individual patient decisions, irrespective of their quoted AUC values. Future research must prioritize calibration as the principal measure of model performance. We strongly urge that studies include both visual calibration plots and quantitative indicators (such as the Brier Score) at the same time to make sure their predictions are accurate in the actual world. Additionally, most of the included studies did not provide reports on the sensitivity and specificity of their models. This affects the overall assessment of model performance, as the reported AUC values may not fully reflect its clinical applicability. Future research should follow standardized reporting guidelines to evaluate its model performance.

External validation is critical to confirm the generalizability of the model’s performance and its applicability to different populations [39,40]. However, all the studies used internal validation methods, such as K-fold cross-validation or random split validation, and only 2 studies conducted external validation. Most of these models lack external validation, further increasing the challenge of clinical implementation. In such a sensitive sector as health care, where predictive models are finally meant to be employed in real-world situations, these ML models may not accurately reflect their applicability and dependability without outside validation [41]. Future studies should incorporate external validation where possible, such as in different hospitals or using publicly accessible databases, to optimize the model generalization through data synthesis and larger datasets [42]. Additionally, we advise temporal validation to assess the model’s resilience at different points in time.

The ML model’s predictive variables found in this study have a number of important effects on clinical practice. First, the predictive factors frequently used in these models play a very important role in current clinical practice and future research. For instance, D-dimer is a well-known clinically validated biomarker for VTE. Studies have shown that it is highly sensitive in predicting the risk of VTE. Prothrombin time, activated partial thromboplastin time, and international normalized ratio are a few other markers that may assist physicians better understand the risk of blood clots and quickly identify and treat patients with high risk [43]. Another established risk factor for VTE is age. Research has indicated that the risk of VTE rises with age. People over the age of 60 years account for around 70% of occurrences of VTE [44]. Considering age while evaluating patients before surgery may enhance outcomes and diminish complication rates. Common chronic disorders, such as diabetes and hypertension, were also among recurring factors. Research has shown that these disorders can raise VTE incidence. A personalized anticoagulant treatment strategy might help those with past histories of these disorders lower their VTE risk even more [45]. Second, the results of the 9 studies on the predictors can guide the development of future predictive models and contribute to later studies on related risk elements. For instance, 1 study [20] included bone cement prosthesis as a predictor in the model, although further study is required to confirm its association with VTE. However, variance in the choosing and reporting of predictors in these studies may affect the comparability and practical utility of the models in different clinical settings.

### Limitations

To our knowledge, this is the first study to evaluate ML in predicting VTE in patients undergoing joint replacement surgery. Nevertheless, the study had certain limitations. First, most of the included studies have been conducted in China and the United States. This geographical concentration restricts the general applicability of the model to different areas. Second, numerous studies focused solely on developing predictive models without considering external validation or practical application of the model. The absence of external validation may affect the credibility of our pooled estimates. Third, since the traditional methods used to assess publication bias (such as funnel plots and Egger or Begg tests) are not applicable to AUC data, no quantitative assessment of publication bias was conducted for the included studies. However, the risk of publication bias remains: “positive” studies (eg, high AUC values) are more likely to be published than “negative” or inconclusive ones. Therefore, the studies included in this review may overestimate the true performance of these models. Finally, the included studies exhibited a high risk of bias and insufficient transparency. These methodological shortcomings could affect the accuracy of model predictions, therefore reducing the dependability and application of the conclusions.

### Conclusions

The study identified 9 studies with a total of 34 ML models for predicting VTE after joint replacement. Most studies have modest to good discriminative ability from their AUC values. However, among these 9 studies, only 2 of them underwent effective external validation. The widespread neglect of external validation has left the generalization capabilities of these models, which performed well on the original dataset, completely unknown. In addition, these studies also show a high risk of bias and significant heterogeneity. Most of the studies had significant methodological shortcomings, including a lack of rigorous study design and absence of calibration measurements. These deficiencies will affect the reliability of the model in clinical applications and reduce the promotion value of the model. To improve the prediction capabilities of ML models, future research must closely follow the PROBAST criteria, which place a strong emphasis on meticulous study design and quality control. External validation is also necessary to improve the generalization and applicability of ML models in clinical settings.

## Supplementary material

10.2196/79886 Multimedia Appendix 1 Details of the search strategy.

10.2196/79886 Checklist 1 PRISMA checklist.

## Abbreviations

AUC: area under the curve
CHARMS: Critical Appraisal and Data Extraction for Systematic Reviews of Prediction
DVT: deep vein thrombosis
LR: logistic regression
ML: machine learning
PE: pulmonary embolism
PRISMA: Preferred Reporting Items for Systematic Reviews and Meta-Analyses
PROBAST: Prediction Model Risk of Bias Assessment Tool
PROSPERO: International Prospective Register of Systematic Reviews
RF: random forest
SVM: support vector machine
THA: total hip arthroplasty
TKA: total knee arthroplasty
VTE: venous thromboembolism
XGB: extreme gradient boosting
