# Supplementary material for: Machine Learning for Predicting Venous Thromboembolism After Joint Arthroplasty: Systematic Review of Clinical Applicability and Model Performance
Source: JMIR Med Inform. 2026 Feb 12;14:e79886. doi: 10.2196/79886 (PMC12900511; doi:10.2196/79886)
Supplement: Multimedia Appendix 1 [file medinform-v14-e79886-s001.docx]

**Supplementary Document:**

**Machine Learning in Predicting Venous Thrombosis Following Joint Arthroplasty：Systematic Review and Meta-Analysis**

Supplementary Table A1- Search Strategy

| **Data Base** | **Search Strategy** |
| --- | --- |
| Pub Med | ((((((((((((((((((((Phlebothrombosis[Title/Abstract]) OR (Thrombosis, Venous[Title/Abstract])) OR (Deep Vein Thrombosis[Title/Abstract])) OR (Thromboses, Deep Vein[Title/Abstract])) OR (Vein Thrombosis, Deep[Title/Abstract])) OR (Deep-Venous Thrombosis[Title/Abstract])) OR (Thrombosis, Deep-Venous[Title/Abstract])) OR (Deep-Vein Thrombosis[Title/Abstract])) OR (Thrombosis, Deep-Vein[Title/Abstract])) OR (Thrombosis, Deep Vein[Title/Abstract])) OR (Deep Venous Thrombosis[Title/Abstract])) OR (Thrombosis, Deep Venous[Title/Abstract])) OR (Venous Thrombosis, Deep[Title/Abstract])) OR (DVT[Title/Abstract])) OR (VTE[Title/Abstract])) OR (deep vein thrombus[Title/Abstract])) OR (Thromboembolism[Title/Abstract])) OR (Thrombosis, Venous[Title/Abstract])) OR (venous thrombosis[MeSH Terms])) AND ((Arthroplasty[MeSH Terms]) OR ((((((Total Knee Arthroplasties[Title/Abstract]) OR (Total Knee Replacements[Title/Abstract])) OR (Knee Replacement Arthroplasties[Title/Abstract])) OR (Total Hip Arthroplasties[Title/Abstract])) OR (Total Hip Replacements[Title/Abstract])) OR (Hip Replacement Arthroplasties[Title/Abstract])))) AND (((((((((((((((((statistic*[Title/Abstract]) OR (data mining[Title/Abstract])) OR (decision tree[Title/Abstract])) OR (random forest[Title/Abstract])) OR (support vector machine[Title/Abstract])) OR (regression[Title/Abstract])) OR (k nearest neighbor[Title/Abstract])) OR (k-mean*[Title/Abstract])) OR (neural network*[Title/Abstract])) OR (deep learning[Title/Abstract])) OR (ensemble learning[Title/Abstract])) OR (predict*[Title/Abstract])) OR (modeling[Title/Abstract])) OR (AI[Title/Abstract])) OR (Artificial Intelligence[Title/Abstract])) OR (risk[Title/Abstract])) OR (machine learning[MeSH Terms])) |
| Web of science | 1: "((((((((((((((TS=(Machine learning)) OR TS=(modeling)) OR TS=(Predict*)) OR TS=(neural network)) OR TS=(artificial intelligence)) OR TS=(AI)) OR TS=(deep learning)) OR TS=(data mining)) OR TS=(decision tree)) OR TS=(random forest)) OR TS=(regression)) OR TS=(Support vector machine)) OR TS=(k nearest neighbor)) OR TS=(k-mean*)) OR TS=(ensemble learning) "  2: "(((((((((TS=(Arthroplasty)) OR TS=(orthopedic surgery)) OR TS=(Total Knee Arthroplasties)) OR TS=(Total Knee Replacements)) OR TS=(Knee Replacement Arthroplasties)) OR TS=(Total Hip Arthroplasties)) OR TS=(Total Hip Replacements)) OR TS=(Hip Replacement Arthroplasties)) OR TS=(TKA)) OR TS=(THA) "  3: "(((((((TS=(deep vein thrombosis)) OR TS=(deep vein thrombus)) OR TS=(Thromboembolism)) OR TS=(Venous Thrombosis)) OR TS=(Phlebothrombosis)) OR TS=(Deep Venous Thrombosis)) OR TS=(DVT)) OR TS=(VTE) "  4:#1 AND #2 AND #3 |
| embase | #8: #3 AND #6 AND #7  #7: 'knee arthroplasty'/exp OR 'hip arthroplasty'/exp  #6: #4 OR #5  #5: dvt:ti,ab,kw  #4: 'vein thrombosis'/exp OR 'thromboembolism'/exp  #3: #1 OR #2  #2: 'machine learning'/exp OR 'artificial intelligence'/exp OR 'deep learning'/exp  #1: : ai:ti,ab,kw OR ml:ti,ab,kw OR 'diagnostic model':ti,ab,kw OR 'prognostic model':ti,ab,kw OR 'prediction model':ti,ab,kw OR 'risk prediction':ti,ab,kw |
| Scopus | (TITLE-ABS-KEY("joint replacement" OR "joint arthroplasty" OR "hip replacement" OR "hip arthroplasty" OR "knee replacement" OR "knee arthroplasty"))  AND  (TITLE-ABS-KEY("machine learning" OR "artificial intelligence" OR "deep learning" OR "neural network*" OR "support vector machine" OR "random forest" OR "predictive model*" OR "prediction model*"))  AND  (TITLE-ABS-KEY("venous thromboembolism" OR "VTE" OR "deep vein thrombosis" OR "DVT" OR "pulmonary embolism" OR "PE")) |
| China National Knowledge Infrastructure (CNKI) | (SU=('关节置换' OR '关节成形' OR '髋关节置换' OR '膝关节置换' OR '人工关节置换'))  AND  (SU=('机器学习' OR '人工智能' OR '深度学习' OR '神经网络' OR '支持向量机' OR '随机森林' OR '预测模型' OR '预测算法'))  AND  (SU=('静脉血栓栓塞' OR 'VTE' OR '深静脉血栓' OR 'DVT' OR '肺栓塞' OR 'PE' OR '血栓')) |
| Wanfang | 主题:("关节置换" OR "关节成形" OR "髋关节置换" OR "膝关节置换" OR "人工关节置换")  AND  主题:("机器学习" OR "人工智能" OR "深度学习" OR "神经网络" OR "支持向量机" OR "随机森林" OR "预测模型" OR "预测算法")  AND  主题:("静脉血栓栓塞" OR "VTE" OR "深静脉血栓" OR "DVT" OR "肺栓塞" OR "PE" OR "血栓") |
| Wipro | (主题='关节置换' OR 主题='关节成形' OR 主题='髋关节置换' OR 主题='膝关节置换' OR 主题='人工关节置换')  AND  (主题='机器学习' OR 主题='人工智能' OR 主题='深度学习' OR 主题='神经网络' OR 主题='支持向量机' OR 主题='随机森林' OR 主题='预测模型' OR 主题='预测算法')  AND  (主题='静脉血栓栓塞' OR 主题='VTE' OR 主题='深静脉血栓' OR 主题='DVT' OR 主题='肺栓塞' OR 主题='PE' OR 主题='血栓') |
